# Alterations of Phosphodiesterases in Adrenocortical Tumors

**DOI:** 10.3389/fendo.2016.00111

**Published:** 2016-08-30

**Authors:** Fady Hannah-Shmouni, Fabio R. Faucz, Constantine A. Stratakis

**Affiliations:** ^1^Program on Developmental Endocrinology and Genetics (PDEGEN), Section on Endocrinology and Genetics (SEGEN), National Institute of Child Health and Human Development (NICHD), National Institutes of Health (NIH), Bethesda, MD, USA

**Keywords:** phosphodiesterases, adrenocortical tumors, adrenal hyperplasia, Cushing syndrome, genetics, Carney complex, cAMP

## Abstract

Alterations in the cyclic (c)AMP-dependent signaling pathway have been implicated in the majority of benign adrenocortical tumors (ACTs) causing Cushing syndrome (CS). Phosphodiesterases (PDEs) are enzymes that regulate cyclic nucleotide levels, including cyclic adenosine monophosphate (cAMP). Inactivating mutations and other functional variants in *PDE11A* and *PDE8B*, two cAMP-binding PDEs, predispose to ACTs. The involvement of these two genes in ACTs was initially revealed by a genome-wide association study in patients with micronodular bilateral adrenocortical hyperplasia. Thereafter, *PDE11A* or *PDE8B* genetic variants have been found in other ACTs, including macronodular adrenocortical hyperplasias and cortisol-producing adenomas. In addition, downregulation of *PDE11A* expression and inactivating variants of the gene have been found in hereditary and sporadic testicular germ cell tumors, as well as in prostatic cancer. PDEs confer an increased risk of ACT formation probably through, primarily, their action on cAMP levels, but other actions might be possible. In this report, we review what is known to date about *PDE11A* and *PDE8B* and their involvement in the predisposition to ACTs.

## Introduction

**Figure 1:**
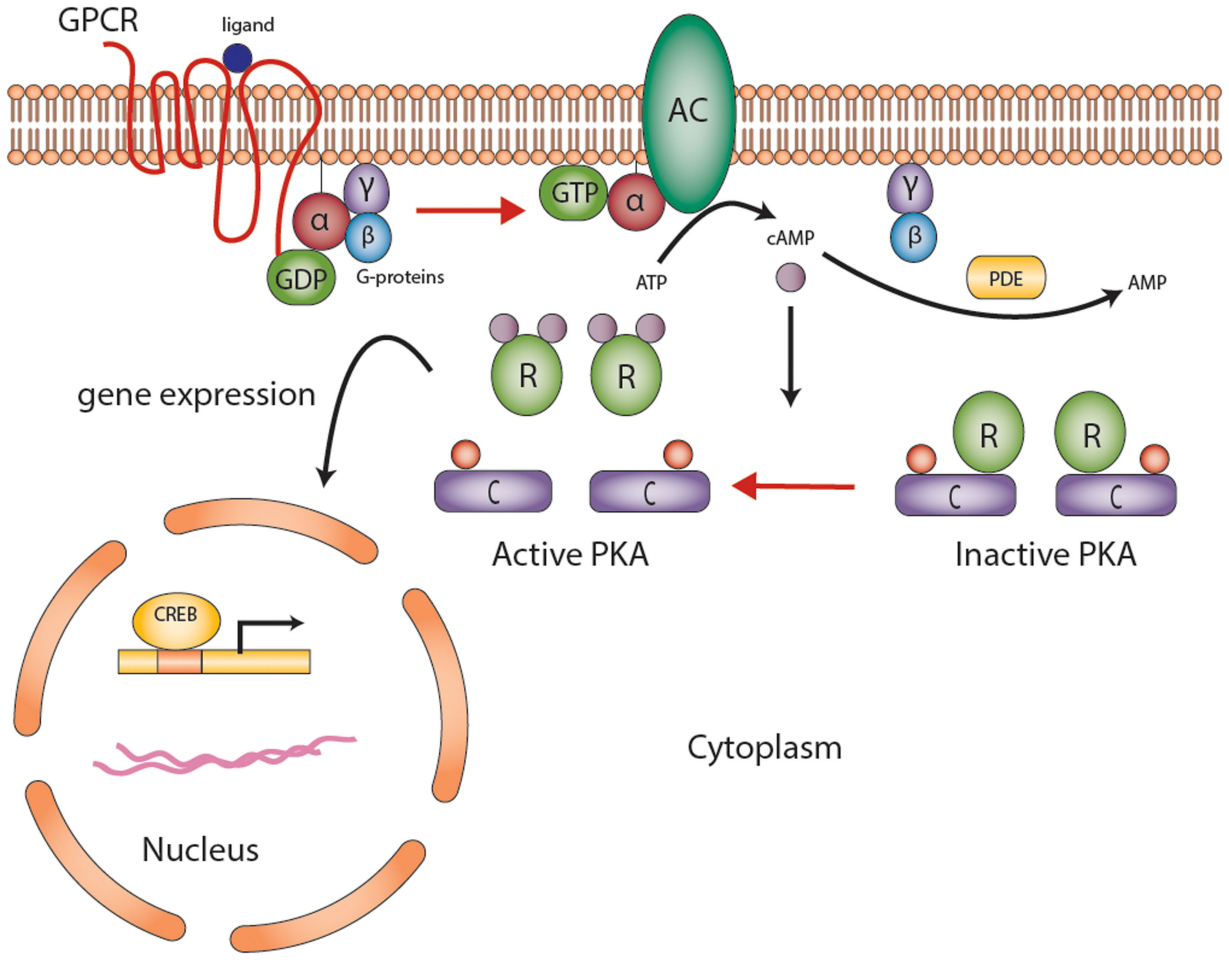
**The cyclic AMP-dependent signaling pathway**. G protein-coupled receptors (GPCRs) undergo conformational changes in response to various extracellular stimuli. Gsα subunit (G-proteins) exchanges GDP for GTP that activates adenylyl cyclase (AC), converting ATP to cAMP. Elevated cAMP levels, regulated by PDEs (also responsible for degradation of cGMP to GMP), then activate protein kinase A (PKA). PKA consists of a tetramer of two homo- or heterodimers regulatory subunits (*R*) and two catalytic subunits (*C*) responsible for the phosphorylation of several enzymes and transcription factors downstream [e.g., cAMP-response element-binding protein (CREB)]. The end product is gene expression to mediate cell growth and differentiation. Abbreviations: α, β, γ, G_s_-protein subunits; AC, adenyl cyclase; C, catalytic subunit of protein kinase A; cAMP, cyclic AMP; CREB, cyclic AMP response element-binding protein, a transcription factor; GPCR, G-protein-coupled receptor; PDE, phosphodiesterase; PKA, protein kinase A; R, the regulatory subunits of protein kinase. Courtesy of Stratakis Lab, NICHD, NIH.

The development and function of the endocrine system is highly dependent on second messengers in hormonal signaling. Advances in molecular and genetic studies and appreciation for the impact of second messengers on endocrine physiology and disease sparked important discoveries in signal pathway research especially over the past decades. The first second messenger to be identified was cyclic adenosine monophosphate (cAMP), described in 1958 by Earl Sutherland (1). The production and degradation of cAMP is regulated by adenyl cyclases (AC) and phosphodiesterases (PDEs), respectively (Figure 1) (1, 2). Recent studies have demonstrated a link between genetic alterations in PDEs and increased predisposition of tumor formation, particularly in the prostate, testis, and the adrenal cortex (3, 4). In primary bilateral macronodular adrenocortical hyperplasia (PBMAH), aberrant expression of several non-mutated G-protein-coupled receptors (GPCRs) showed that cAMP signaling could be increased without genetic mutations (5). In primary pigmented nodular adrenocortical disease (PPNAD), germline inactivating mutations of the protein kinase A regulatory subunit type 1 (*PRKAR1A*)-linked cAMP-dependent protein kinase (PKA) to adrenocortical tumors (ACTs) (6). Indeed, cAMP signaling dysregulation through expression defects or mutations appears to underlie the pathogenesis of most benign ACTs (5, 7).

Phosphodiesterases exist in over 100 isoforms and are derived from 21 genes separated into 11 *PDE* gene families (summarized in Table 1) (8–10). PDEs function through the hydrolyzation of cAMP (PDEs isoform 4, 7, and 8) and cyclic guanosine monophosphate (cGMP) (PDEs isoforms 5, 6, and 9) into AMP and GMP, respectively (8, 9). Dual-specificity PDEs (acting on both cAMP and cGMP with varying affinities) include the PDE1, PDE2, PDE3, PDE10, and PDE11 enzymes. All PDEs share a major structural feature; a conserved catalytic domain with about 300 amino acids located near the C-terminal regions, and a variable regulatory domain located in the N-terminal regions. PDEs vary in a number of ways, including a difference in substrate selectivity, tissue distribution, kinetic, and tissue expression (Table 1). The adrenal cortex expresses several isoforms of PDEs, including PDE2A that has been implicated in the downregulation of aldosterone production in adrenal zona glomerulosa cells and the regulation of the adrenocorticotropic hormone (ACTH)-induced increase in intracellular cAMP in the zona fasciculata cells (11).

**Table 1:** **Characteristics of phosphodiesterases (PDEs)**.

PDEs	Gene(s)	Locus	Substrate	Major functions/regulations
PDE1	*PDE1A*	2q32.1	cAMP > cGMP	Vascular smooth muscle contraction, sperm function Dopaminergic signaling, immune cell activation Vascular smooth muscle cell proliferation, sperm function
	*PDE1B*	12q13.2	cAMP > cGMP	
	*PDE1C*	17p14.3	cAMP = cGMP	
PDE2	*PDE2A*	11q13.4	cAMP = cGMP	Aldosterone and ACTH secretion, long-term memory
PDE3	*PDE3A*	12p12.2	cAMP > cGMP	Cardiac contractility, platelet aggregation, vascular smooth muscle contraction, oocyte maturation, and regulation of renin release Impact on lipolysis, glycogenolysis, insulin secretion, and cardiac function
	*PDE3B*	11p15.2		
PDE4	*PDE4A*	19p13.2	cAMP	Brain function, monocyte and macrophage activation, neutrophil infiltration, vascular smooth muscle proliferation, fertility Regulate β-adrenergic signaling and excitation–contraction coupling in the heart and thus play a role in vasodilatation and cardiac contractility
	*PDE4B*	1p31.3		
	*PDE4C*	19p13.11		
	*PDE4D*	5q11.2–q12.1		
PDE5	*PDE5A*	4q26	cGMP > cAMP	Modulate NO/cGMP effects in vascular smooth muscles, platelets, and lower urinary tract organs Cardiac stress response
PDE6	*PDE6A*	5q32	cGMP > cAMP	Primary effector enzyme in the phototransduction cascade Regulate cGMP concentration in rod and cone photoreceptors
	*PDE6B*	4p16.3		
	*PDE6C*	10q23.33		
PDE7	*PDE7A*	8q13.1	cAMP	Play a critical role in the regulation of the human T-cells function
	*PDE7B*	6q23.3		
PDE8	*PDE8A*	15q25.3	cAMP	Play a role in T-cell activation Regulate adrenal steroidogenesis Regulate TSH levels Control of LH signaling and steroidogenesis in Leydig cells
	*PDE8B*	5q13.3		
PDE9	*PDE9A*	21q22.3	cGMP > cAMP	Energy balance
PDE10	*PDE10A*	6q27	cAMP > cGMP	Play a role in striatal activation and behavioral activity
PDE11	*PDE11A*	2q31.2	cAMP = cGMP	Only the A4 splice variant is expressed in adrenal tissue Sperm production

*ACTH, adrenocorticotropic hormone; cAMP, cyclic adenosine monophosphate; cGMP, cyclic guanine monophosphate; LH, luteinizing hormone; PDE11A, phosphodiesterase 11A gene; NO, nitric oxide; TSH, thyroid-stimulating hormone*.

*Adapted from Ref. (10)*.

Recent studies have demonstrated inactivating mutations and other germline variants in *PDE11A* and *PDE8B* in ACT causing Cushing syndrome (CS) (12–17). Here, we present a brief overview of the alterations of PDEs in ACTs. Given the breadth of this topic, we begin with a discussion of the cAMP-dependent signaling pathway in physiology, describe the current classification of ACTs, and then proceed with a discussion of PDE alterations in ACTs.

## The cAMP-Dependent Signaling Pathway

**Figure 2:**
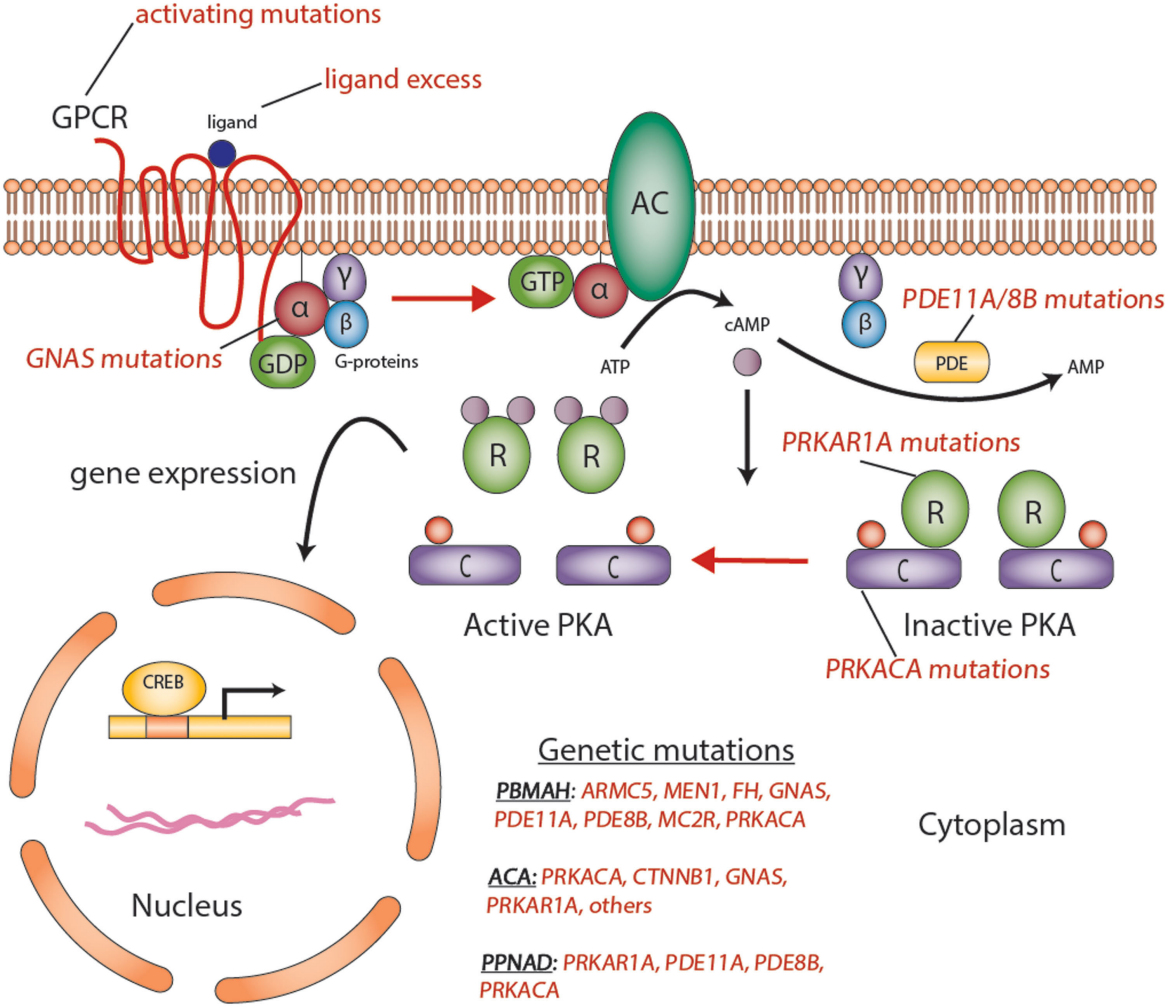
**Aberrations in the cyclic AMP-dependent signaling pathway in adrenocortical tumors**. Mutations such as activating mutations of *GNAS*, in McCune–Albright syndrome; inactivating germline mutations in *PDE11A* in primary pigmented nodular adrenocortical hyperplasia (PPNAD); and inactivating germline mutations of *PRKAR1A* in Carney’s complex, all predispose to the formation of adrenocortical tumors through a cyclic AMP-dependent process. Abbreviations: α, β, γ, G_s_-protein subunits; AC, adenyl cyclase; ACA, adrenocortical adenoma; C, catalytic subunit of protein kinase A; cAMP, cyclic AMP; CREB, cyclic AMP response element-binding protein, a transcription factor; GPCR, G-protein-coupled receptor; PBMAH, bilateral macronodular adrenocortical hyperplasia; PDE, phosphodiesterase; PKA, protein kinase A; R, the regulatory subunits of protein kinase; PPNAD, primary pigmented nodular adrenocortical disease. Courtesy of Stratakis Lab, NICHD, NIH.

Briefly, GPCRs undergo conformational changes in response to various extracellular stimuli, such as ACTH (Figure 1). The first step in cAMP activation in adrenal cortex is the action of ACTH on its seven-transmembrane receptor, ACTHR [e.g., melanocortin 2 receptor (MC2R)]. This activation leads to the dissociation of the Gsα subunit (encoded by the *GNAS* gene) from the heterotrimeric G-proteins, activation of AC, generation of cAMP, and activating PKA (Figure 1). PKA exists as a tetrameric complex of two regulatory subunits (alpha and beta type 1 or alpha and beta type 2, encoded by *PRKAR1A, PRKAR2A, PRKAR1B*, and *PRKAR2B*) and two catalytic subunits (catalytic alpha and catalytic beta, encoded by *PRKACA* and *PRKACB*, respectively); the latter subunits are responsible for the phosphorylation of several enzymes and transcription factors downstream, including the cAMP-response element-binding protein (CREB). Abnormalities in some of these genes predispose to the formation of cortisol-producing ACTs (Figure 2) and increased steroid hormone secretion. The latter is often mediated by secondary factors regulated by PKA; in one study, silencing p54(nrb)/NONO expression in H295R human adrenocortical cells decreased the ability of the cells to increase intracellular cAMP production and subsequent cortisol biosynthesis in response to ACTH (18). The expression of multiple PDE isoforms, including PDE2A, PDE3A, PDE3B, PDE4A, PDE4D, and PDE11A, was induced in p54(nrb)/NONO knockdown cells, which suggests that these proteins may be responsible for the splicing and degradation of *PDE* transcripts (18). Studies in knockout mice (Table 2) have also pointed out to the significance of cAMP signaling-regulating genes in ACT formation.

**Table 2:** **Adrenocortical phosphodiesterases mouse models and phenotypes**.

Gene	Model	Phenotype
*PDE2*	Pde2a^tm1Dgen^	Homozygous for knockout allele exhibit lethality early in gestation therefore difficult to study (58)
	Pde2a^tm1Dtst^	
*PDE8*	Pde8b^tm1Dgen^	Homozygous for null allele with increased urine corticosterone, decreased serum ACTH, and decreased sensitivity to a PDE8-selective inhibitor (39)
*PDE11*	Pde11a^tm1Lex^	No adrenal phenotype. Homozygous for null allele have reduced sperm concentration (45)

## A Historical Perspective and Nomenclature of Adrenocortical Tumors

In 1912, Cushing described the pituitary tumors that cause the condition that today bears his name (19). The causes of CS are broadly divided into ACTH-dependent and ACTH-independent disease. In 1984, the first description of Carney complex, a multiple neoplasia syndrome associated with spotty skin pigmentation, cardiac myxomas, pituitary tumors, and CS caused by PPNAD, provided the first insight into the genetic forms of ACT (20). In 1991, Weinstein et al. (21) described Gsα subunit (*GNAS*) mutations in individuals with McCune-Albright syndrome (MAS); MAS is classically associated with polyostotic fibrous dysplasia, café-au-lait skin spots, and precocious puberty but also with adrenal hyperplasia and/or tumors (21). MAS, albeit rare, appears to be the most frequent cause of CS among ACTH-independent adrenal hyperplasia in the infantile period (22). Kirschner et al. (23, 24) identified the regulatory subunit type 1A (R1α) of PKA (encoded by the *PRKAR1A* gene on chromosome 17q22-24) as the cause of PPNAD and CNC (25). PPNAD is the most frequent endocrine manifestation in CNC.

In 2006, the first association between *PDE* mutations and ACT, using a genome-wide association (GWA) study approach, was identified in patients with CS with PPNAD but without *GNAS* or *PRKAR1A* mutations (12). Three inactivating mutations in *PDE11A* were identified initially in patients, predominantly children, with micronodular adrenocortical hyperplasia (iMAD), a rare form of bilateral adrenocortical hyperplasia (BAH) leading to CS. Subsequent studies found other *PDE* mutations or functional variants, including alterations in *PDE8B*, in other ACTs, including PBMAH (14–17, 26, 27).

The new genetic findings influenced the diagnostic classification of these ACTs (5). In brief, three major ACTH-independent ACT subtypes exist: bilateral hyperplasias (BAH), adrenocortical adenomas (ACA), and adrenocortical cancer (ACC) (5). PBMAH, a form of BAH, is estimated to affect ~2% of patients with endogenous CS (5). This disease should be distinguished from secondary adrenocortical hyperplasia, which can occur after long-term stimulation by ACTH in Cushing disease (CS in the context of an ACTH-producing pituitary tumor) or ectopic ACTH secretion, predominantly from a neuroendocrine tumor (28). PBMAH is usually sporadic, but familial forms have been described (29). Cortisol-producing adenoma (CPA) is a benign subset of ACAs causing adrenal CS.

Bilateral adrenocortical hyperplasias can be broadly classified on the basis of the size of their nodules into micronodular (<1 cm in diameter) or macrocronodular (>1 cm in diameter) (5). The micronodular subtype is divided into pigmented (c-PPNAD, familial seen in CNC, or isolated, i-PPNAD) and not pigmented (e.g., iMAD) (5). PBMAH is the most common macronodular BAH [previously referred to as massive macronodular adrenocortical disease (MMAD) and ACTH-independent macronodular adrenocortical hyperplasia (AIMAH)] and is largely caused by mutations of the *ARMC5* gene (30); several genes have been implicated in other forms of macronodular BAH, including *GNAS, APC*, and *MEN1*. Patients with MAS may develop a form of macronodular bilateral adrenal hyperplasia, called primary bimorphic adrenocortical disease (PBAD).

## PDEs in Adrenocortical Tumors

Mutations and variants in ACTs that lead to functional abnormalities of cAMP signaling have been reported in the *GNAS, PRKAR1A, PDE11A*, and *PDE8B* genes (9). Mutation-negative disease with activation of the cAMP pathway has been reported (31), suggesting that additional genetic (or perhaps epigenetic) “hits” may play a role in the pathogenesis of ACT.

### PDE2A

The predominant PDE isoform in adrenal tissue is PDE2A (32). Three PDE2A isoforms exist: PDE2A1, PDE2A2, and PDE2A3, and exhibit higher affinity for cGMP than cAMP (33). PDE2A is implicated in the downregulation of aldosterone production in adrenal zona glomerulosa cells and the regulation of the ACTH-induced increase in intracellular cAMP (11). This ACTH response is described as a rapid and sustained activation of AC followed by a biphasic effect of ACTH on PDE2 activity with an initial and rapid inhibition, followed by a delayed activation (11). In one study, PDE2 involvement was observed to be more important in rat than in human adrenal glomerulosa cells, whereas AC was more stimulated in human than in rat glomerulosa cells (34). Thus, PDE2 activity is involved in the regulation of cAMP accumulation induced by ACTH and suggests that ACTH inhibits this activity. However, no studies to date have reported an association between alterations in PDE2 and ACTs. PDE2A has been shown to be upregulated in beta-catenin (*CTNNB1*)-mutated ACTs (35). However, it has not been studied in individuals with PPNAD that also have somatic mutations in *CTNNB1* (36, 37). Importantly, *Pde2a* knockout mice do not survive past 17–18 days gestation (38); *Pde2a* heterozygote mice are not known to develop ACTs or even hypertension.

### PDE8

The PDE8 family of proteins includes two genes, *PDE8A* and *PDE8B*, which encode for two highly specific enzymes responsible for the highest affinity of the PDEs to degrade cAMP (32). Through negative modulation, these isomers play an important role in adrenal, ovarian, and testicular steroidogenesis (32, 39). Although homologous in structure and function, *PDE8B* is the major regulator of one or more pools of cAMP in steroidogenesis and carries the highest expression across zona fasciculata compared to other PDEs (14, 32, 39). PDE8A is expressed from a small population of zona fasciculata cells that lie adjacent to zona glomerulosa (32), while *PDE8B* is expressed throughout the zona fasciculata. The *PDE8B* locus, like that of other PDEs, is quite complex and encodes multiple isoforms, arising mainly from alternative splicing and displaying tissue-specific expression (32, 40, 41). Tsai et al. (39) demonstrated that *Pde8b* knockout mice showed elevated urinary corticosterone as a result of adrenal hypersensitivity toward ACTH (39), pointing to PDE8B’s possible role in regulating steroidogenesis. However, the investigators also demonstrated that these mice do not develop adrenal hyperplasia or increased adrenal size.

A GWA study identified a link between the 5q13 locus harboring the *PDE8B* gene and iMAD (12). A novel missense mutation was found in *PDE8B* (c.914A > C, p.P305H) in a 2-year-old girl with iMAD where her father carried the same genetic defect with subclinical disease (15); this pattern of an unaffected male passing on the disease to an affected female was also seen in other alterations of PDEs (12, 42). The p.P305H mutation led to higher levels of cAMP when introduced in HEK293 cells (15).

A more recent study of ACTs found several variations in *PDE8B*: missense substitutions p.H391A, p.P660L, and p.V697I and the c.1365-5G>A splice variant in *PDE8B* were identified (43). Interestingly, one patient with ACC had both the missense p.R121H and the splice c.1365-5G>A variations, while the other germline *PDE8B* mutations were found in samples including PBMAH, PPNAD, and secreting and even non-secreting ACAs (43).

Perhaps, the most important recent finding confirming PDE8B’s role in ACT pathogenesis was the genome-wide transcriptomic work by Wilmot Roussel et al. (44). Among over 3000 genes that showed correlation with cortisol secretion in 22 unilateral ACAs (5 non-secreting, 6 subclinical cortisol producing, and 11 cortisol producing), *PDE8B* showed the strongest positive correlation (44). Accordingly, there was marked increase of the PKA activity to cAMP ratio in secreting adenomas compared to non-secreting adenomas (44).

### PDE11A

*PDE11A* is located on chromosome 2q31.2 and encodes a dual-specificity PDE that degrades both cAMP and cGMP (32). This gene is highly polymorphic in the general population (42) and was the first of the PDEs to be linked with an inherited condition associated with ACTs. Four different transcript variants exist (Table 1), with only the *PDE11A4* detected in adrenal tissues (32). *Pde11a* knockout mice show impaired sperm function and spermatogenesis (45), but no adrenal phenotype has been described for the knock out or heterozygote mouse. Several studies have reported conflicting results with regard to the adrenal expression of PDE11A, suggesting that its expression may only be driven in the diseased adrenal gland (46, 47). The exact role of *PDE11A* in regulating adrenocortical cAMP levels also remains largely unknown.

Horvath et al. (12) published the first GWA single-nucleotide polymorphism (SNP) association between *PDE11A* mutations and ACTs in patients with CS from PPNAD or iMAD without known genetic defects (12). Three inactivating mutations in *PDE11A* were identified. These tumors showed 2q31–2q35 loss of heterozygosity (LOH) and elevated cAMP levels (12), supporting *PDE11A*’s role in tumor formation. Three of the four patients had PPNAD; a mother and her daughter with the same *PDE11A* gene mutation, and a third unrelated patient with a different *PDE11A* mutation, in which the adrenal glands were described as small (largest total adrenal weight = 6.9 g and normal = 8–9 g) with very minor involvement of the superficial cortex evidenced by a few transcapsular cortical extensions into the peri-adrenal fat (48). The fourth patient’s adrenal glands were slightly enlarged owing to hyperplasia of the superficial cortex with a few PPNAD-type nodules in the deep cortex (48). The *PDE11A* mutation was inherited from her father who had an enlarged right adrenal gland but no CS (48).

In another study, Horvath et al. (42) examined two relatively frequent variants of *PDE11A* in ACTs and the general population (42). Twelve of 745 controls had these variants, with a lower frequency in patients with ACTs (1.6%; χ^2^ = 14.62, *P* < 0.0001). *In vitro* data demonstrated elevated cAMP levels in HeLa and HEK293 cells, particularly when the p.R804H mutation was studied (42). Another study showed the p.R867G *PDE11A* gene variant in one patient with familial PBMAH (49). The mechanism by which partially inactivated *PDE11A* causes adrenocortical overgrowth is largely unclear; the most likely explanation is chronic (albeit modest) elevations of cAMP levels in adrenocortical tissues. Collectively, these experiments suggest that genetic variations in *PDE11A* may be low-penetrance alleles that occur relatively frequently in the general population and may predispose to the development of ACTs.

The association of *PDE11A* variants and ACTs was studied further in larger cohorts. Libé et al. (27) examined the role of the *PDE11A* in a large cohort of ACT, and found an inactivating mutation (p.R307*) in one ACC, with a significant difference between ACC and controls for a polymorphism in exon 6 (p.E421E; OR, 2.1; *P* = 0.03) (27). Three associated polymorphisms located in intron 10–exon 11–intron 11 were also significant in these tumors (OR, 0.5; *P* = 0.01) (27). Other variants in the study included 22 germline missense variants (18.8%) in ACA, compared to only 11 missense variants (5.7%) in controls (16 versus 10% in ACC, 19 versus 10% in ACA, and 24 versus 9% in PBMAH; OR, 3.53; *P* = 0.05) (27). This study suggested a higher frequency of mutations in ACTs, especially PBMAH, when compared to controls. In another study that examined a large cohort of patients with PBMAH, the frequency of all *PDE11A* variants (e.g., p.D609N or p.M878V) was significantly higher among patients with PBMAH (28%) than controls (7.2%) (*P* = 5 × 10^−5^) (17). These variants were also studied in HEK293 cells, where the mutant *PDE11A*-transfected cells had higher cAMP levels than the wild-type ones (*P* < 0.05), suggesting that these mutants exhibit diminished cAMP hydrolytic activity (17).

These experiments pointed to several important points about the possible *PDE11A*’s role in adrenocortical tumorigenesis. First, the spectrum of ACTs varies from benign to malignant. Second, bilateral disease is favored. Third, the allelic losses of the wild-type allele in ACC with missense mutations supports *PDE11A* role as a tumor suppressor gene. Fourth, *PDE11A* sequence defects may underlie at least part of the commonly found adrenocortical incidentalomas.

Alterations in PDEs may also be involved in modifying the expression of syndromic diseases associated with ACTs. CNC is caused by *PRKAR1A* mutations, as discussed earlier (23). In one study of 150 patients with CNC, a higher frequency of *PDE11A* variants was observed when compared with healthy controls (25.3 versus 6.8%, *P* < 0.0001), particularly in men (30.8 versus 13%, *P* = 0.025, PPNAD subgroup) (16). Importantly, these men had a higher incidence of large-cell calcifying Sertoli cell tumors, as well (16). Moreover, simultaneous *in vitro* inactivation of *PRKAR1A* and *PDE11A* by small inhibitory RNA led to increased PKA activity and/or cAMP signaling (16). Thus, it is conceivable that *PDE11A*-inactivating variants act in concert with other genes in disease predisposition and/or progression.

## Aberrations in the cAMP-Dependent Signaling Pathway in Cortisol-Producing Adenomas

Several genetic aberrations in the cAMP-dependent signaling pathway have been implicated in CPA. The most common genetic aberration in CPA is a somatic-activating mutations of *PRKACA* (c.617A>G/p.L206R) with an estimated incidence of ~42% (86 of 206 tumors studied to date) (50–52), with a predilection to younger patients with overt CS, suggesting a driver mutation role in tumorigenesis (44). Somatic mutations in *GNAS* were identified in 5–17% of CPA (53). The somatic allelic losses of *PRKAR1A* were described in 23% of CPA; these tumors were smaller in size and had a paradoxical increase in urinary cortisol levels after dexamethasone suppression (54), due to increased glucocorticoid receptor expression in ACT (55), as often observed in patients with c-PPNAD. Defects in *Wnt*-signaling have been reported in CPA, with *CTNNB1* (p.S45P, p.S45F) in ~23% of cases (56). There are still many unknown genetic defects that lead to CPA formation.

## Future Directions

There has been significant progress in PDE-related research over the past two decades. Genetic testing has uncovered several adrenocortical conditions that were linked to aberrations in *PDE*, often preceded by a long and insidious pre-diagnostic course. This has allowed earlier identification and better management of these lesions. However, there are several unanswered questions. PDE-related research is hampered by inherent (i.e., complexity of their structures, many intracellular interactions, and largely unknown function) and technical issues (e.g., there is lack of specific antibodies for the multiple isoform of each PDE). In addition to improving the characterization of PDE expression and function, future studies should also focus on the characterization of patients with various ACT phenotypes and PDE genotypes.

Preliminary results in using recombinant compounds to activate or inhibit the PDE11A structure may have important implications for drug development. Jäger et al. (47) produced approximately fourfold to fivefold increase in PDE11A-mediated hydrolysis of both cAMP and cGMP, with some degree of PDE11A specificity, with a cGMP analog (Rp-8-pCPTPET-cGMPS) bound to the PDE11A4 GAF domain (47). Furthermore, Ceyhan et al. (57) showed that BC11-28 and BC11-38 (potent and selective PDE11A inhibitors) in both yeast-based and enzyme assays had a >350-fold selectivity for inhibiting PDE11’s cGMP hydrolytic activity versus all other PDEs, while only BC11-38 inhibited PDE11A cAMP hydrolytic activity in H295R cells (57). Thus, a targeted molecular therapy approach for lesions related to defects in PDE may aid in the future management of affected or at risk patients.

## Conclusion

Alterations in PDEs that lead to dysregulation of the cAMP-dependent signaling pathway have been linked to the development of ACT. These lesions are usually benign and represent an important group of genetic disorders causing CS. As genetic technology continues to revolutionize the field of endocrine genetics and as we continue to discover novel disease-causing genes on an unprecedented scale, new methods to rapidly assess the functional significance of *PDE* variants singly, or in combination, will evolve. In this review, we focused our discussion on the various genotypes and phenotypes of ACT due to alterations in *PDE*, particularly mutations in *PDE8B* and *PDE11A*. Although there has been significant progress in PDE-related research over the past two decades, there are as yet unidentified molecular causes for all of these lesions. We hope that one day targeted molecular therapies will replace adrenalectomy as the treatment of choice for these lesions.

## Author Contributions

All authors contributed equally to the conception or design of the work; or the acquisition, analysis, or interpretation of data for the work; drafting the work or revising it critically for important intellectual content; final approval of the version to be published; agreement to be accountable for all aspects of the work in ensuring that questions related to the accuracy or integrity of any part of the work are appropriately investigated and resolved.

## Conflict of Interest Statement

The authors declare that the research was conducted in the absence of any commercial or financial relationships that could be construed as a potential conflict of interest.

## Abbreviations

AC, adenyl cyclase; ACTH, adrenocorticotropic hormone; AIMAH, ACTH-independent macronodular adrenal hyperplasia; AMP/ATP, adenosine monophosphate/adenosine triphosphate; Alleles, alternative forms of a gene; cAMP, cyclic adenosine monophosphate; CNC, Carney complex; CS, Cushing syndrome; CREB, cAMP-response element-binding protein; GMP/GDP/GTP, guanosine monophosphate/guanosine diphosphate/guanosine triphosphate; Genes, units of inheritance at specific locations (loci) on a chromosome; GPCRs, G protein-coupled receptors; GWA, genome-wide association; heterozygous, a genotype with two different alleles of a gene for a particular trait; homozygous, a genotype with the same allele of a gene for a particular trait; MMAD, massive macronodular adrenocortical disease; mutations, alteration of genetic material producing a new variation; PBAD, primary bimorphic adrenocortical disease; PBMAH, primary bilateral macronodular adrenocortical hyperplasia; PDEs, phosphodiesterases; phenotype, detectable expression of a genotype; PKA, protein kinase A; PPNAD, primary pigmented micronodular adrenal disease; PRKAR1A, protein kinase A regulatory subunit type 1.
